# Effects of *S. mutans* gene-modification and antibacterial calcium phosphate nanocomposite on secondary caries and marginal enamel hardness

**DOI:** 10.1039/c9ra09220j

**Published:** 2019-12-17

**Authors:** Hong Chen, Yunhao Tang, Michael D. Weir, Lei Lei, Radi Masri, Christopher D. Lynch, Thomas W. Oates, Ke Zhang, Tao Hu, Hockin H. K. Xu

**Affiliations:** State Key Laboratory of Oral Diseases, Department of Preventive Dentistry, West China Hospital of Stomatology, Sichuan University Chengdu Sichuan 610041 China hutao@scu.edu.cn; Department of Advanced Oral Sciences and Therapeutics, University of Maryland Dental School Baltimore MD 21201 USA hxu@umaryland.edu tuzizhangke@163.com; Second Affiliated Hospital of Chongqing Medical University Chongqing 400010 China; Restorative Dentistry, University Dental School and Hospital, University College Cork Wilton Cork Ireland; School of Stomatology, Capital Medical University Beijing China; Center for Stem Cell Biology & Regenerative Medicine, University of Maryland School of Medicine Baltimore MD 21201 USA; Marlene and Stewart Greenebaum Cancer Center, University of Maryland School of Medicine Baltimore MD 21201 USA

## Abstract

Secondary caries at the restoration-tooth margins is a main reason for dental restoration failures. Gene-modification for *Streptococcus mutans* (*S. mutans*) and composites containing dimethylaminohexadecyl methacrylate (DMAHDMA) and nanoparticles of amorphous calcium phosphate (NACP) all have the potential to suppress bacterial acids and promote remineralization. However, there has been no report of their effects on marginal caries-inhibition and enamel hardness. The objective of this study was to investigate the effects of gene-modification and DMAHDM–NACP composite restoration on enamel demineralization and hardness at the margins under biofilm acids for the first time. Parent *S. mutans* and *rnc* gene-deleted *S. mutans* were tested side by side. The bioactive composite contained 3% DMAHDM and 30% NACP. Mechanical properties and calcium (Ca) and phosphate (P) ion releases were measured. Colony-forming units (CFU), MTT, lactic acid and polysaccharide of biofilms were evaluated. Demineralization of bovine enamel with composite restorations was induced *via* biofilms, then enamel hardness was measured. The dual strategy of combining *rnc*-deletion with DMAHDM+30NACP: (1) achieved the strongest biofilm-inhibition, with the greatest reduction in biofilm CFU by 6 logs; (2) decreased biofilm lactic acid and polysaccharide production by more than 80%; (3) achieved enamel hardness that was 140% higher than that of a commercial fluoride-releasing composite under 30 days of biofilm acids. Therefore, the novel dual approach of *rnc* gene-deletion and DMAHDM+NACP nanocomposite is promising to inhibit secondary caries at the margins and increase the longevity of tooth restorations.

## Abstract 2

Secondary caries at the restoration-tooth margins is a main reason for dental restoration failures.

## Graphical abstract

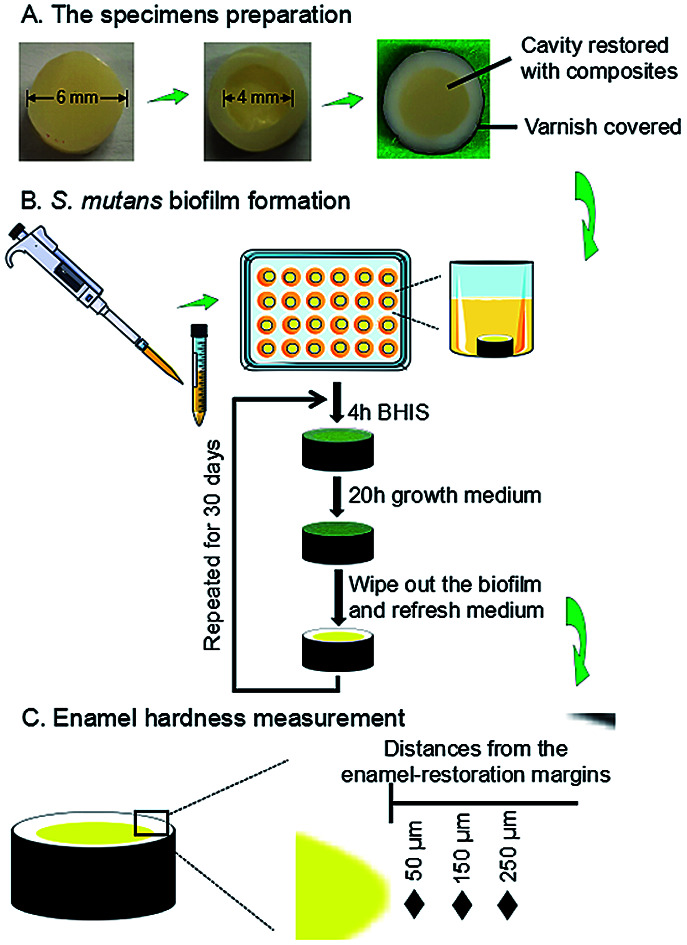

## 1. Introduction

Resin composites have become a popular restorative material for tooth cavity restorations due to their excellent aesthetics, minimally invasive preparation, direct-filling technique, and the remarkable improvements in their mechanical properties.^1–6^ However, studies showed that resin composites had thicker biofilm formation and higher secondary caries incidence, compared to other restorative materials such as amalgams and glass ionomer cements.^7–9^ Secondary (recurrent) caries adjacent to the restoration margins was reported as one of the most frequent causes of restoration failures.^1,9–11^ Therefore, it would be highly desirable to develop a new generation of therapeutic composites with antibacterial and remineralization capabilities to tackle secondary caries.^12,13^

Quaternary ammonium methacrylates (QAMs) are cationic antimicrobials with a wide spectrum of activities.^14–19^ QAMs can be co-polymerized and covalently bonded in dental resins, thus achieving long-term antibacterial effects.^14–19^ Previous studies developed several novel QAMs and incorporated them into dental resins, including 12-methacryloyloxydodecylpyridinium bromide (MDPB),^16,17^ quaternary ammonium polyethylenimine (QPEI) nanoparticles,^18,19^ quaternary ammonium methacryloxy siliane molecule (QAMS; C44H90ClNO18Si5),^15^ methacryloxylethyl cetyl dimethyl ammonium chloride (DMAE-CB),^15^ 2-dimethyl-2-dodecyl-1-methacryloxyethyl ammonium iodine (DDMAI),^20,21^ dimethylaminododecyl methacrylate (DMADDM)^22^ and dimethylaminohexadecyl methacrylate (DMAHDM).^14,15^ DMAHDM had an alkyl chain length of 16 and was used to provide antibacterial activity through the contact-killing mechanism.^14,23,24^ Furthermore, calcium phosphate (CaP)-releasing resins were also developed for caries-inhibition to suppress demineralization and promote remineralization.^25–27^ Nanoparticles of amorphous calcium phosphate (NACP) with a mean size of 116 nm were synthesized *via* a spray-drying technique.^28,29^ NACP nanocomposite released high levels of calcium (Ca) and phosphate (P) ions to neutralize acids and protect tooth structures.^30^ The novel multifunctional DMAHDM–NACP nanocomposite displayed both remineralization and potent antibacterial capabilities, inhibiting biofilm viability and lactic acid production.^13,31^

*Streptococcus mutans* (*S. mutans*) is a principle cariogenic pathogen.^32^*S. mutans* can effectively utilize dietary sucrose to synthesize exopolysaccharides (EPS),^33^ which act as a multifunctional scaffold for biofilm growth and contribute to the recalcitrance pathogenicity, environmental stress tolerance, and antimicrobial resistance.^34–36^ Recently, the genes and pathways of *S. mutans* targeting the EPS matrix were found to play an important role in the interactions with anti-caries compounds.^37,38^ The *rnc* gene (SMU_1514), encoding the ribonuclease III (RNase III), is a post-transcriptional regulator gene located in the upstream of the VicRKX tricistronic operon.^39,40^ It was revealed that the deletion of the *rnc* gene beneficially inhibited the production of EPS matrix, suppressed the biofilm formation, and decreased the cariogenicity by repressing the downstream VicRKX expression at the post-transcriptional level.^39,40^ In addition, targeting EPS network disrupted the viscoelastic properties to further weaken biofilm cohesiveness and enhance the antimicrobial efficacy.^41^ Indeed, recent studies demonstrated that EPS synthesis inhibitors and EPS-degrading enzymes enhanced the antimicrobial delivery and killing efficacy of antibiotics and antimicrobial peptides.^42,43^ However, to date, there has been no report on the effects of *S. mutans rnc* gene-modification together with the DMAHDM–NACP nanocomposite on the inhibition of secondary caries for tooth cavity restorations.

Compare to planktonic bacteria, biofilm is more resistant to antimicrobials and more clinically significant for caries research.^34,44,45^ A number of biofilm models have been developed to assess the killing effects of antibacterial materials and techniques against caries-pathogenic bacteria and their effects on caries formation in enamel and dentin.^9,46,47^ These *in vitro* models are meritorious for standardized control and accurate measurements during the pre-defined time periods.^48,49^ In the oral environment, there is a dynamic equilibrium between demineralization and remineralization at the tooth-pellicle and plaque–saliva interfaces in the caries process.^50^ Measurement of enamel hardness was used in assessing the enamel demineralization and remineralization.^51,52^ Enamel hardness mapping adjacent to the restoration was used to reveal information on enamel demineralization level around the margin.^51,52^ To date, there has been no report on the use of a biofilm model to determine the effects of bacterial gene-modification and DMAHDM–NACP nanocomposite on enamel hardness around the restoration margins.

Therefore, the objective of the present study was to investigate the effects of gene-modification and DMAHDM–NACP composite restoration on enamel demineralization and hardness at the margins under biofilm acids for the first time. It was hypothesized that: (1) the *S. mutans rnc* gene-deletion alone would reduce the biofilm growth and EPS synthesis; (2) DMAHDM–NACP composite would greatly reduce biofilm growth and acid production; (3) the combination of *S. mutans rnc* gene-deletion with DMAHDM–NACP composite would protect the tooth structures at the restoration margins under biofilms, yielding much greater enamel hardness than control groups.

## 2. Materials and methods

### 2.1. Fabrication of composites

Bisphenol glycidyl dimethacrylate (BisGMA, Esstech, Essington, PA, USA) and TEGDMA (Esstech, Essington, PA, USA) were mixed at a 1 : 1 mass ratio. Then, 0.2% camphorquinone and 0.8% ethyl 4 *N*,*N*-dimethylaminobenzoate for light-cure. This resin is referred to as BT resin.^53^ DMAHDM was incorporated into BT at a DMAHDM/(BT+DMAHDM) mass fraction of 3%, following a previous study.^23^ Barium boroaluminosilicate glass particles of a median diameter of 1.4 μm (Caulk/Dentsply, Milford, DE, USA) were silanized with 4% 3-methacryloxypropyltrimethoxysilane and 2% *n*-propylamine (all mass%).^23^ A mass fraction of 70% of glass particles was mixed with 30% BT resin, yielding a cohesive paste.

A spray-drying technique was used to prepare the NACP, as previously described.^28,54^ Briefly, calcium carbonate and dicalcium phosphate anhydrous were dissolved into an acetic acid solution, and the final Ca and P ionic concentrations were 8 mmol L^−1^ and 5.333 mmol L^−1^, respectively. The solution was sprayed into a heated chamber to evaporate the water and volatile acid. The dried particles were collected *via* an electrostatic precipitator (Air Quality, Minneapolis, MN). This yielded NACP with a mean particle size of 116 nm.^28^

A commercial fluoride-releasing nanocomposite (Heliomolar, Ivoclar, Amherst, NY, USA) was used as a comparative control. Heliomolar contained nanoparticles of sizes of 40–200 nm of silica and ytterbium–trifluoride, at a filler level mass fraction of 66.7%.^55^ Seven composites were tested for mechanical properties:

(1) Heliomolar nanocomposite (referred to as commercial control);

(2) Experimental composite control. 30% BT + 70% glass particles (referred to as BT+glass control);

(3) Antibacterial composite. 27% BT + 70% glass + 3% DMAHDM + 0% NACP (referred to as DMAHDM+0NACP);

(4) Antibacterial and remineralizing composite. 27% BT + 60% glass particles + 3% DMAHDM + 10% NACP (referred to as DMAHDM+10NACP);

(5) Antibacterial and remineralizing composite. 27% BT + 50% glass particles + 3% DMAHDM + 20% NACP (referred to as DMAHDM+20NACP);

(6) Antibacterial and remineralizing composite. 27% BT + 40% glass particles + 3% DMAHDM + 30% NACP (referred to as DMAHDM+30NACP);

(7) Antibacterial and remineralizing composite. 27% BT + 30% glass particles + 3% DMAHDM + 40% NACP (referred to as DMAHDM+40NACP).

Composite disks were made using molds with a diameter of 10 mm and a thickness of 1 mm, which were light-cured (Triad 2000; Dentsply, York, PA, USA) for 1 min.^23^ The cured disks were immersed in deionized water at 37 °C and agitated for 24 hours to remove any initial burst of uncured monomers, following a previous study.^23^ The disks were sterilized with ethylene oxide (Anprolene AN 74i, Andersen, Haw River, NC, USA) and degassed for 3 days, following the manufacturer's instructions.^23,56^

### 2.2. Mechanical testing

Each composite paste was placed into rectangular molds of 2 × 2 × 25 mm. Each sample was covered in Mylar strips and light-cured (Triad 2000, Dentsply, York, PA) for 1 min on each open side of the mold.^5,57^ The specimens were stored at 37 °C for 24 hours and then placed on three-point flexure with a 10 mm span at a crosshead-speed of 1 mm min^−1^ on a computer-controlled Universal Testing Machine (5500R, MTS, Cary, NC).^58^ The flexural strength was: *S* = 3*P*_max_/*L*(2*bh*^2^), where *P*_max_ is the fracture load, *L* is span, *b* is specimen width and *h* is thickness. Elastic modulus was: *E* = (*P*/*d*)(*L*^3^/[4*bh*^3^]), where load *P* divided by displacement *d* is the slope in the linear elastic region.

The mechanical testing showed that the flexural strength decreased significantly when the mass fraction of NACP exceeded 30%. Therefore, the following four groups were used for subsequent experiments: commercial control; BT+glass control; DMAHDM+0NACP; DMAHDM+30NACP.

### 2.3. Bacterial strains and growth conditions

The use of all bacteria strains was approved by the University of Maryland Baltimore Institutional Review Board Institutional Review Board. The parent *S. mutans* strain UA159 (ATCC 700610), and the *rnc*-deleted strain Smurnc were provided by the State Key Laboratory of Oral Diseases (Sichuan University, Chengdu, China).^39,40^ The parent *S. mutans* were cultured in the brain-heart infusion (BHI) broth (Sigma, St. Louis, MO, USA) without antibiotics, and incubated at 37 °C with 5% CO_2_. For the *rnc*-deleted *S. mutans*, 10 μg mL^−1^ erythromycin was included in the BHI medium.^39,40^

For the biofilm assays, each sterilized composite disk was placed into a well of 24-well plates. The overnight parent and *rnc*-deleted *S. mutans* was adjusted to 10^7^ colony-forming units (CFU) per mL in 1.5 mL of culture medium without antibiotics for each well, using BHI supplemented with 2% sucrose (wt/vol) (BHIS).^59^ The bacteria concentrations of the inoculum were determined using a spectrophotometer (Genesys 10S, Thermo Scientific, Waltham, MA, USA). After 24 hours, composite disks with biofilms were transferred to new 24-well plates filled with fresh BHIS. Then the cultures were incubated at 37 °C with 5% CO_2_ for another 24 hours. This totaled two days of culture which formed relatively mature biofilms on resins.

### 2.4. Ca and P ion release from NACP nanocomposites

A sodium chloride (NaCl) solution (133 mmol L^−1^) was buffered to pH 4 with 50 mmol L^−1^ lactic acid, and pH 7 with 50 mmol L^−1^ HEPES. One specimen of 2 × 2 × 12 mm was immersed in 15 mL of solution at each pH, yielding a specimen volume/solution of 3.2 mm^3^ mL^−1^. This was similar to a specimen volume per solution of approximately 3.0 mm^3^ mL^−1^ in a previous study.^60^ Six specimens were tested and averaged for ion release for each group. For each solution, the concentrations of Ca and P released from the specimens were measured at 1, 3, 7, 14, 21, 28, 35, 42, 49, 56, 63 and 70 days. At each time, aliquots of 0.5 mL were removed and replaced by fresh solution.^54^ The aliquots were analyzed for Ca and P ion concentrations *via* a spectrophotometric method (SpectraMax M5, Molecular Devices) using known standards and calibration curves.^54^ The released ions were reported in cumulative ion concentrations.^28,61^

### 2.5. Live/dead bacteria assay

Disks with 48 h biofilms were washed with cysteine peptone water (CPW), then stained with BacLight Live/Dead bacterial viability kits (Molecular Probes, Eugene, OR, USA).^23^ A mixture of 2.5 μM SYTO 9 and 2.5 μM propidium iodide was set on each sample for 15 minutes. The live bacteria were stained by SYTO 9 to a green color, and the compromised bacteria were stained by propidium iodide into a red color. Images were captured with an inverted epifluorescence microscope (TE2000-S, Nikon, Melville, NY, USA). Three disks were used for each group and each disk was tested in five random positions, yielding 15 images per group.

### 2.6. Biofilm CFU counts

Six disks of each group with 48h biofilms were used. The biofilms were harvested in PBS by scraping and sonication/vortexing (Fisher, Pittsburg, PA, USA). The suspensions were serially diluted and spread on BHI plates. After 48 hours incubation at 37 °C in 5% CO_2_, the colony number was counted and CFU counts were determined.^26^

### 2.7. MTT metabolic assay of biofilms

The 3-(4,5-dimethylthiazol-2-yl)-2,5-diphenyl tetrazolium bromide (MTT) metabolic (VWR Chemicals, Ohio, USA) assay was used.^23^ Disks with 48h biofilms were washed twice with PBS and transferred into new 24-well plates (*n* = 6). One mL MTT dye (0.5 mg mL^−1^ MTT in PBS) was added into each well and incubated for 1 h at 37 °C in 5% CO_2_.^23,62^ Then the disks were transferred into new 24-well plates with 1 mL dimethyl sulfoxide (DMSO) in each well and incubated at room temperature for 20 min to dissolve the formazan crystals. The DMSO solution was then transferred into 96-well plate, and OD_540nm_ was determined using a microplate reader (SpectraMax M5, Molecular Devices, Sunnyvale, CA, USA). Six disks were tested for each group.

### 2.8. Polysaccharide synthesis by biofilms

Water-insoluble polysaccharides of biofilms were measured using the phenol-sulfuric acid method.^14,23^ Six disks biofilms at 48 hours were tested for each group. Biofilms on disks were collected by scraping and sonication/vortexing in 2 mL PBS. The precipitate was then washed twice with PBS, and then they were centrifuged (12 000 rpm) for 5 min at 4 °C. The water-insoluble polysaccharides were washed twice with PBS, and resuspended in 200 μL distilled water. 200 μL of 5% phenol solution and 1 mL of 95–97% sulfuric acid were added, then incubation at room temperature for 30 min. After mixing by pipetting, 200 μL of the solution was transferred into a 96-well plate and OD_490nm_ was determined with the microplate reader (SpectraMax M5, Molecular Devices).

### 2.9. Lactic acid production by biofilms

Disks with 48h biofilms were washed twice with PBS, then immersed in 1.5 mL buffered peptone water (BPW, Sigma-Aldrich) supplemented with 0.2% sucrose and incubated at 37 °C in 5% CO_2_ for 3 hours (*n* = 6).^55^ The lactate concentrations in BPW were determined using a lactate dehydrogenase enzymatic method by measuring OD_340nm_, and the concentrations were determined using the lactic acid standard curves, as previously described.^62^

### 2.10. *In vitro S. mutans* biofilm-enamel demineralization model

**Fig. 1:**
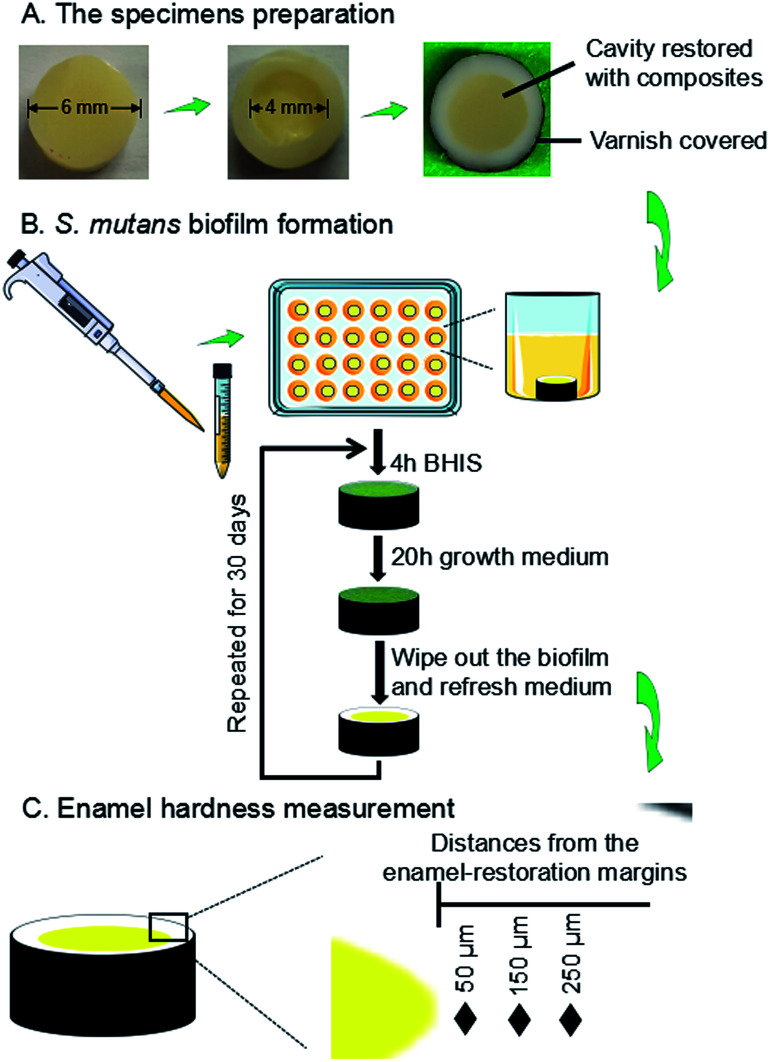
Schematic diagram illustrating the workflow of experimental design. This included an *S. mutans* biofilm model with composite restoration in enamel. (A) Sample preparation, (B) biofilm formation, demineralization, and (C) enamel hardness measurements.

The use of freshly extracted and intact bovine incisors was approved by the Institutional Review Board of University of Maryland Baltimore. As shown in Fig. 1A, enamel slabs were prepared to have a diameter of 6 mm and a thickness of 2.5 mm. Circular cavities with an approximate diameter of 4 mm and a depth of 1.5 mm were prepared. The rest of the enamel surfaces were coated with two layers of acid-resistant nail varnish, except a circle of 1 mm width around the cavity, where enamel was exposed to face biofilms to test demineralization later. The specimens were polished using sandpapers with grits of # 600, 1200, 2400 and 4000, consecutively, with copious water.

A two-way full factorial design was tested: two types of bacteria (parent *S. mutans*, and *rnc*-deleted *S. mutans*), and four composites as described below. This led to eight groups: (1) parent *S. mutans* and commercial control; (2) parent *S. mutans* and BT+glass control; (3) parent *S. mutans* and DMAHDM+0NACP; (4) parent *S. mutans* and DMAHDM+30NACP; (5) *rnc*-deleted *S. mutans* and commercial control; (6) *rnc*-deleted *S. mutans* and BT+glass control; (7) *rnc*-deleted *S. mutans* and DMAHDM+0NACP; (8) *rnc*-deleted *S. mutans* and DMAHDM+30NACP.

The enamel slabs were randomly divided into eight groups. Each group of 6 slabs were restored with a composite. After 24 hours, the slabs were polished again and sterilized with ethylene oxide (Anprolene AN 74i, USA). The sterile specimens were placed in 24-well plates containing 1.5 mL of *S. mutans* suspension, as previously described.^63^ Each day, the slabs was placed into a well of 24-well plates, and immersed in 1.5 mL of BHIS at pH 7.4 at 37 °C for 4 hours. Then it was removed and placed into a new plate with the growth medium at pH 7 for 20 hours at 37 °C in 5% CO_2_.^63–65^ There were enough P ions in the BHI medium. Ca ions and PIPES buffer were added in the solution.^64,65^ The growth medium contained 3.7 g L^−1^ BHI, 25 mM PIPES buffer (acid form) and 1.5 mM CaCl_2_. The pH of growth medium was adjusted to 7.0 by the addition of KOH (5 M). The daily medium change was done under aseptic conditions. Since this biofilm model took 30 days to finish, to avoid the biofilm becoming old and too thick which would deprive the interior bacteria from nutrients, a sterile paper was used to remove the biofilm from the slab every 24 hours, which was then inoculated again to grow a new biofilm on the slab. This biofilm model and cyclic immersion treatment was repeated for 30 days (Fig. 1B).

### 2.11. Enamel hardness measurement near the restoration margins

A hardness tester (HMV II; Shimadzu Corporation, Kyoto, Japan) was used with a Vickers indenter, under a 50 g load with a dwell time of 20 s (Fig. 1C).^66,67^ The area selected for indentation was the enamel surface located at three distances (50 μm, 150 μm, 250 μm) from the margin of the restored cavity, following previous studies.^68,69^ These distances covered the interfacial enamel area near the restoration margins known to be prone to secondary caries.^68,69^ Every enamel specimen had six indentations made at each of the aforementioned three distances, with six specimens for each group, yielding 108 indents per group. Measurements were conducted before and after biofilm acid attacks. Because the baseline enamel hardness (before biofilm attack) at three distances in different enamel samples had only minor differences, they were all pooled together to provide one mean value and standard deviation for sound enamel hardness.

### 2.12. Statistical analysis

Data analyses were conducted using the statistical software SPSS 19.0 (SPSS, Chicago, IL, USA). The normal distribution assumption and Levene's homogeneity tests were confirmed for the variables. One-way and two-way analyses of variance (ANOVA) were applied to detect the significant effects of the variables. Tukey's multiple comparison tests were performed. The significance level was set at a *p* value of 0.05.

## 3. Results

### 3.1. Flexural strength and elastic modulus

**Fig. 2:**
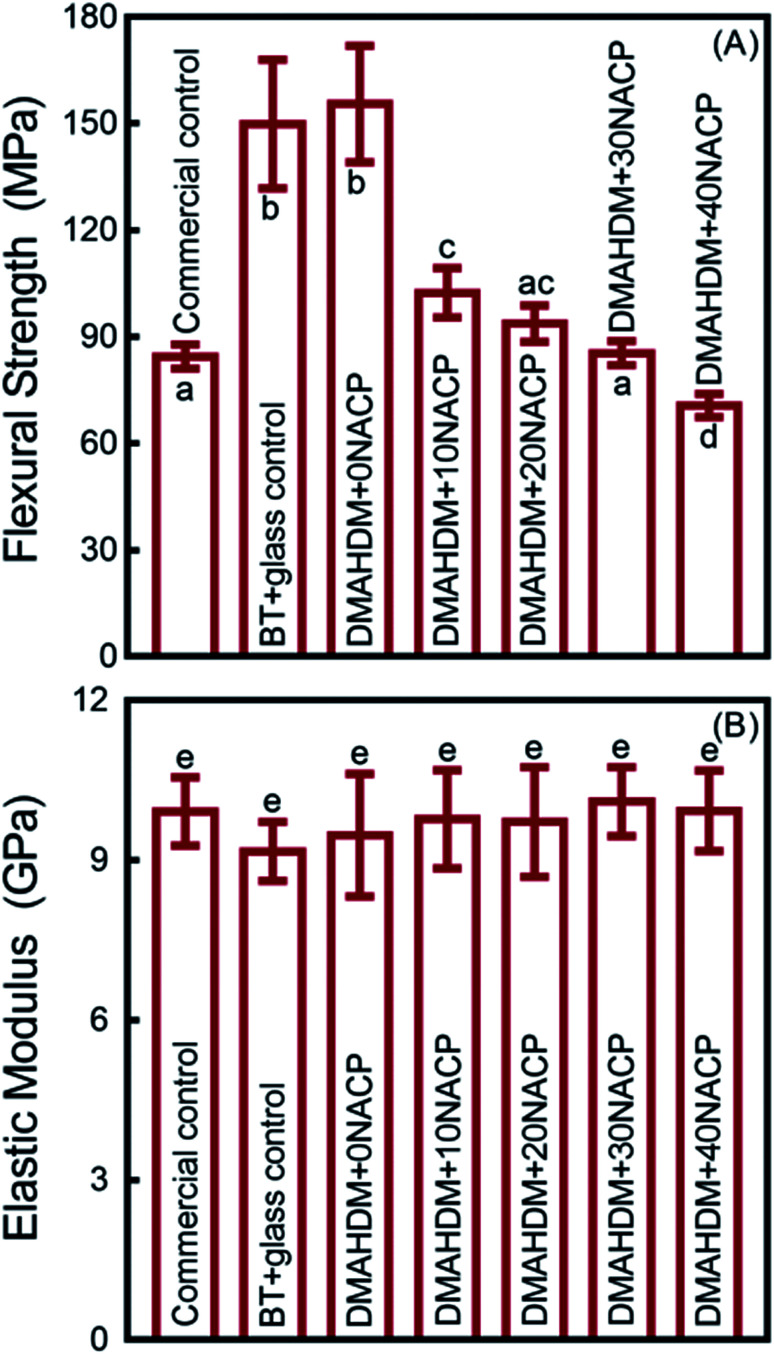
Mechanical properties of composites (mean ± sd; *n* = 6). (A) Flexural strength, and (B) elastic modulus. Values with dissimilar letters are significantly different from each other (*p* < 0.05).

The mechanical properties of the composites are plotted in Fig. 2 (mean ± standard deviation (sd); *n* = 6). BT+glass control and DMAHDM+0NACP group had the highest flexural strength, and they were significantly higher than the commercial fluoride-releasing Helimolar composite control (*p* < 0.05) (Fig. 2A). With increasing the mass fraction of NACP from 0% to 40%, the flexural strength had a decreasing trend. The 40% NACP significantly reduced the flexural strength (*p* < 0.05), compared to the Helimolar (Fig. 2A). For the elastic modulus, the mean values for all the groups ranged between 9.16 to 10.02 GPa (Fig. 2B). All the groups had similar elastic modulus (*p* > 0.05).

### 3.2. Ca and P ion release

**Fig. 3:**
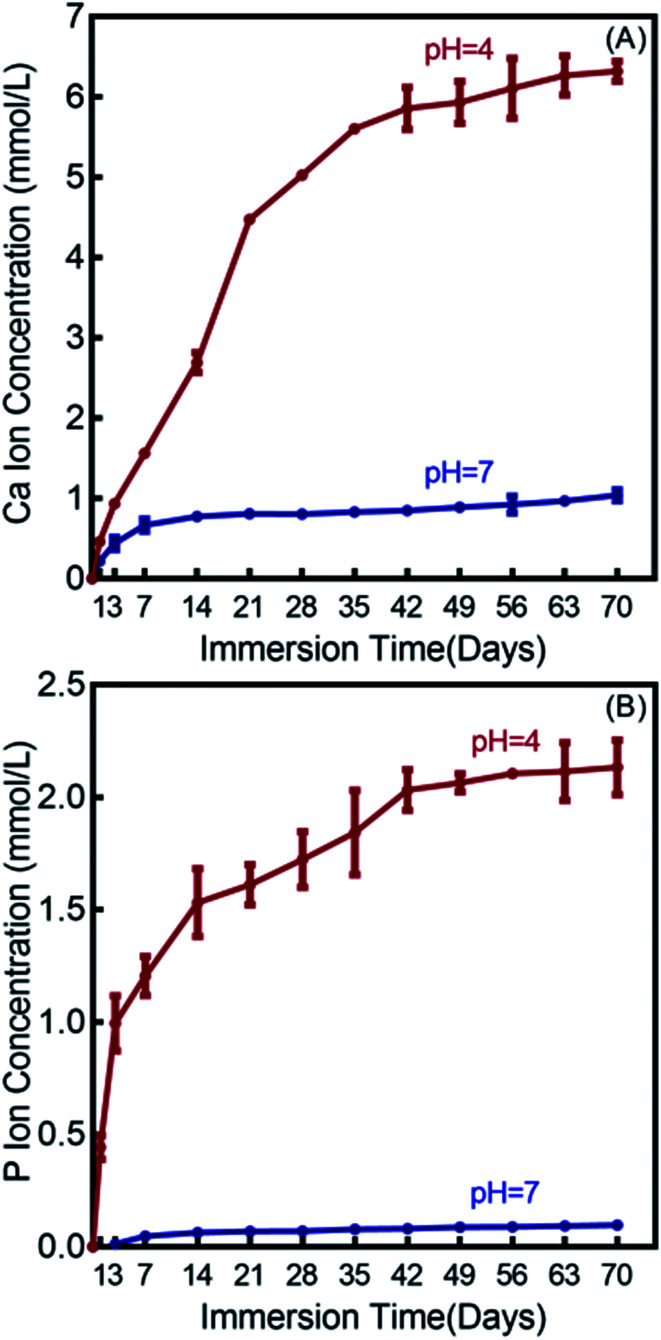
Calcium (Ca) and phosphate (P) ion releases from the DMAHDM+30NACP composite immersed in solutions of pH 4 and 7 (mean ± sd; *n* = 6). (A) Ca ion release, and (B) P ion release.

The Ca and P ion release concentrations from DMAHDM+30NACP composite are plotted in Fig. 3 (mean ± sd; *n* = 6). The composites in the other three groups had no NACP with no Ca and P release. The Ca and P ion releases were much faster in the pH 4 solution than that in the pH 7 solution. After 70 days, the total accumulative Ca ion concentration was 6.32 mmol L^−1^ in the pH 4 solution and 1.04 mmol L^−1^ in the pH 7 solution. The released P ion concentrations were 2.13 mmol L^−1^ and 0.10 mmol L^−1^ in pH 4 and pH 7 solutions, respectively.

### 3.3. Live/dead assay for biofilms grown on composites

**Fig. 4:**
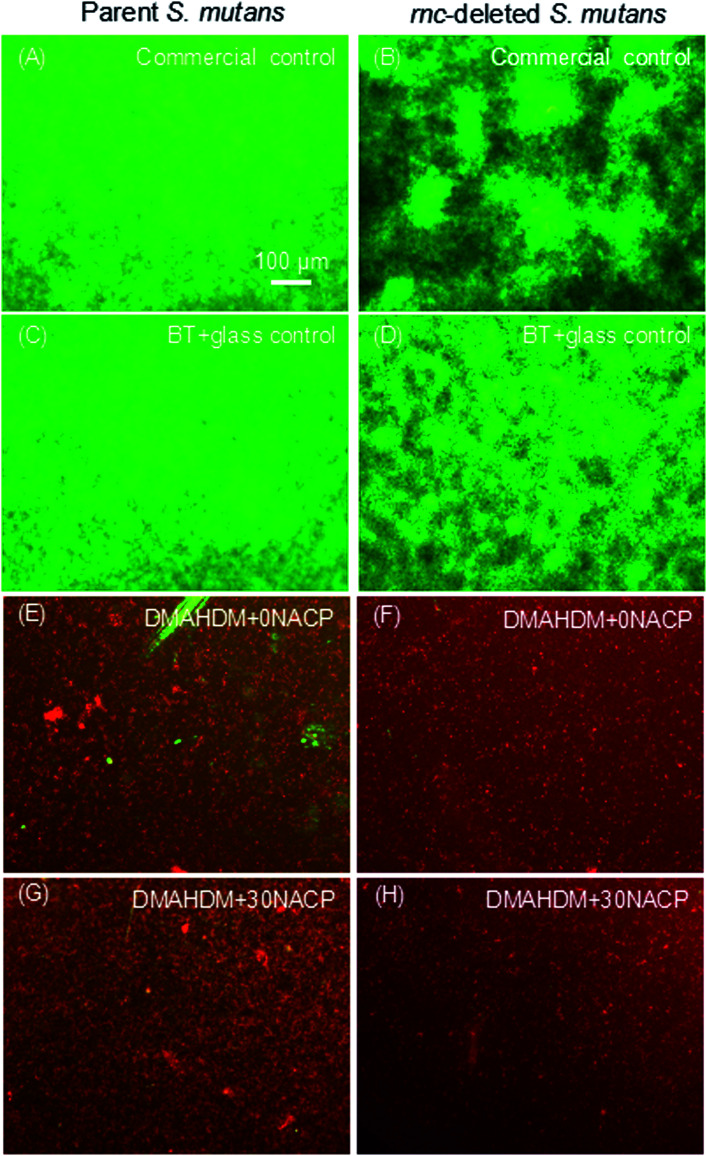
Representative live/dead staining images of 2 day biofilms of parent and *rnc*-deleted *S. mutans* on composites. (A and B) Commercial control; (C and D) BT+glass control; (E and F) DMAHDM+0NACP; (G and H) DMAHDM+30NACP.

Fig. 4 showed representative live/dead images of 48 h biofilms on the composites. Commercial control and BT+glass control had full coverage of primarily live bacteria. In contrast, the biofilms on DMAHDM+0NACP and DMAHDM+30NACP consisted of mainly compromised bacteria.

### 3.4. Antibacterial effects of composites

**Fig. 5:**
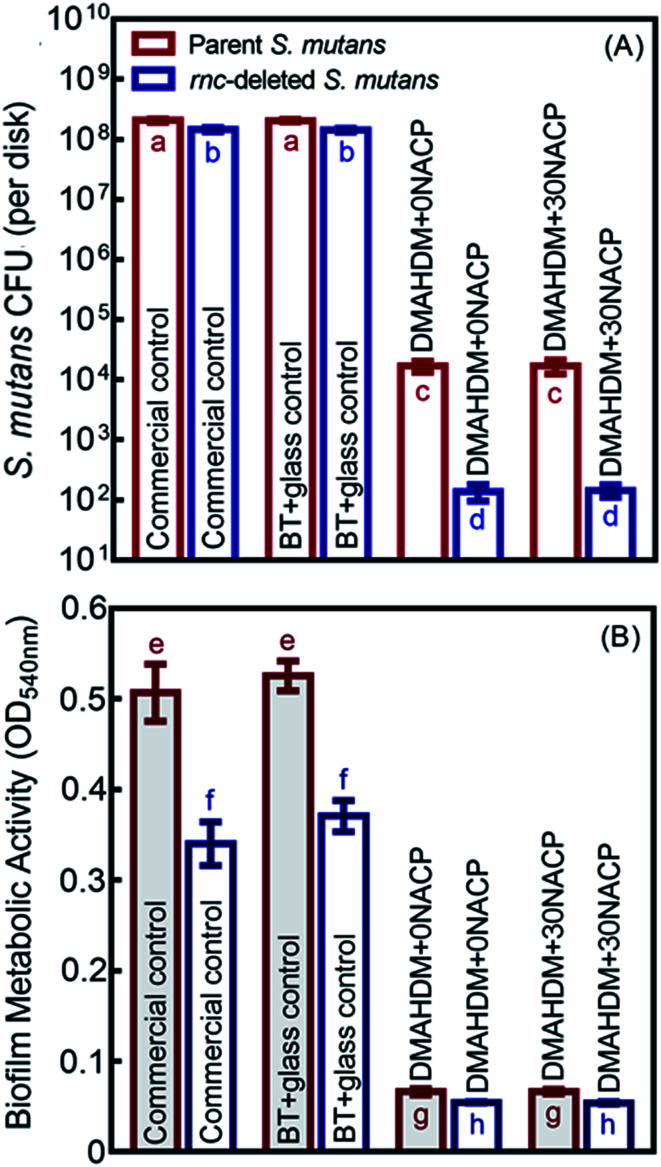
Antibacterial effects of composites on the parent and *rnc*-deleted *S. mutans* biofilms (mean ± sd; *n* = 6). (A) Colony-forming units (CFU), and (B) MTT metabolic activity, of *S. mutans* biofilms on composites. In each plot, values with dissimilar letters are significantly different from each other (*p* < 0.05).

As shown in Fig. 5A, the 48 h biofilm CFU (mean ± sd; *n* = 6) for commercial control and BT+glass control were more than 10^8^ CFU per disk. For the parent *S. mutans*, the CFU counts of the DMAHDM+0NACP and DMAHDM+30NACP were decreased to 10^4^ CFU per disk (*p* < 0.05). In addition to the effects of *rnc* gene deletion, biofilm CFU values were substantially decreased to 10^2^ CFU per disk in the *rnc*-deleted *S. mutans* biofilm (*p* < 0.05). Similarly, Fig. 5B indicates that DMAHDM+0NACP and DMAHDM+30NACP yielded much lower metabolic activity of biofilms than control (*p* < 0.05). Furthermore, the combination of *rnc* gene-deletion and DMAHDM+30NACP achieved the lowest metabolic activity of biofilms (*p* < 0.05).

### 3.5. Inhibition of cariogenic activities

**Fig. 6:**
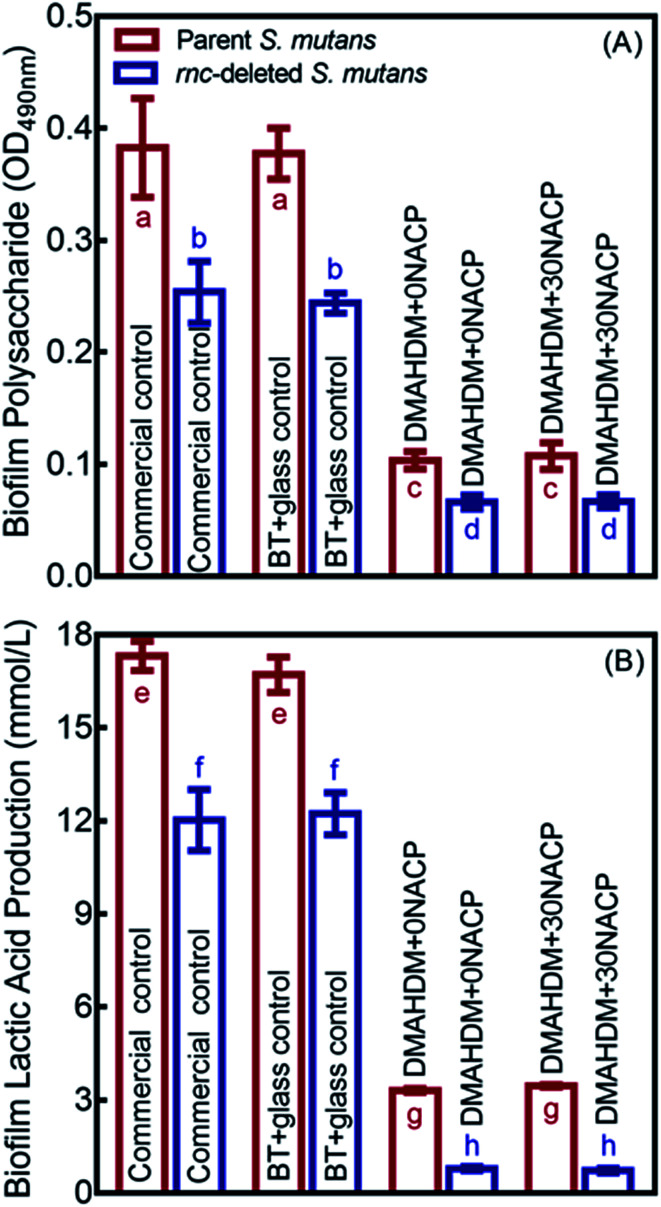
Inhibition effects of composites against cariogenic activities of the parent and *rnc*-deleted *S. mutans* biofilms. (A) Polysaccharide synthesis by *S. mutans* biofilms on composites (mean ± sd; *n* = 6). (B) Lactic acid production by *S. mutans* biofilms (mean ± sd; *n* = 6). In each plot, values with dissimilar letters are significantly different from each other (*p* < 0.05).

Polysaccharide and lactic acid productions of 48 h biofilms on composites are showed in Fig. 6 (mean ± SD; *n* = 6). The deletion of *rnc* remarkably decreased the polysaccharide synthesis and lactic acid production, compared to the parent *S. mutans* (*p* < 0.05). In addition, DMAHDM+0NACP and DMAHDM+ 30NACP composites greatly reduced polysaccharide and lactic acid production, compared to control composites (*p* < 0.05). Furthermore, using the dual strategy, the combination of *rnc* deletion with DMAHDM–NACP composite achieved the greatest reduction in polysaccharide and lactic acid productions.

### 3.6. Enamel hardness testing

**Fig. 7:**
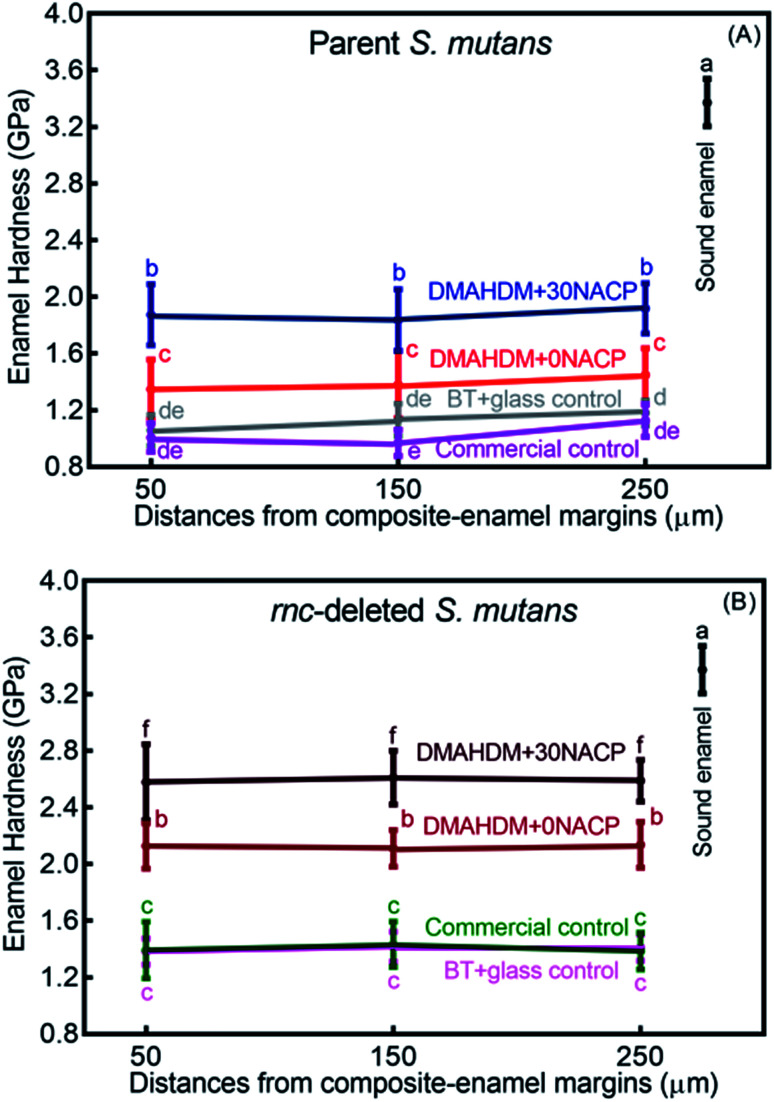
Enamel hardness at three distances from the margin of the composite restoration after 30 days of acid production treatment in biofilm model (mean ± sd; *n* = 36). (A) Parent *S. mutans* and (B) *rnc*-deleted *S. mutans*. Values with dissimilar letters are significantly different from each other (*p* < 0.05).

Enamel hardness around the restoration margins is plotted in Fig. 7, at three different distances in enamel from the cavity restoration interface (mean ± sd; *n* = 36). The sound enamel hardness was 3.37 ± 0.17 GPa. After 30 days under the parent or *rnc*-deleted *S. mutans* biofilm acid attack, enamel hardness at different distances all decreased, but the DMAHDM+30NACP group had the highest enamel hardness (*p* < 0.05). For example, at a distance of 50 μm, under parent *S. mutans* biofilm, the enamel hardness was 1.87 ± 0.22 GPa for DMAHDM+30NACP, which was significantly higher than 1.35 ± 0.21 GPa for DMAHDM+0NACP, 1.06 ± 0.11 GPa for BT+glass, and 1.01 ± 0.10 GPa for commercial control (*p* < 0.05).

**Fig. 8:**
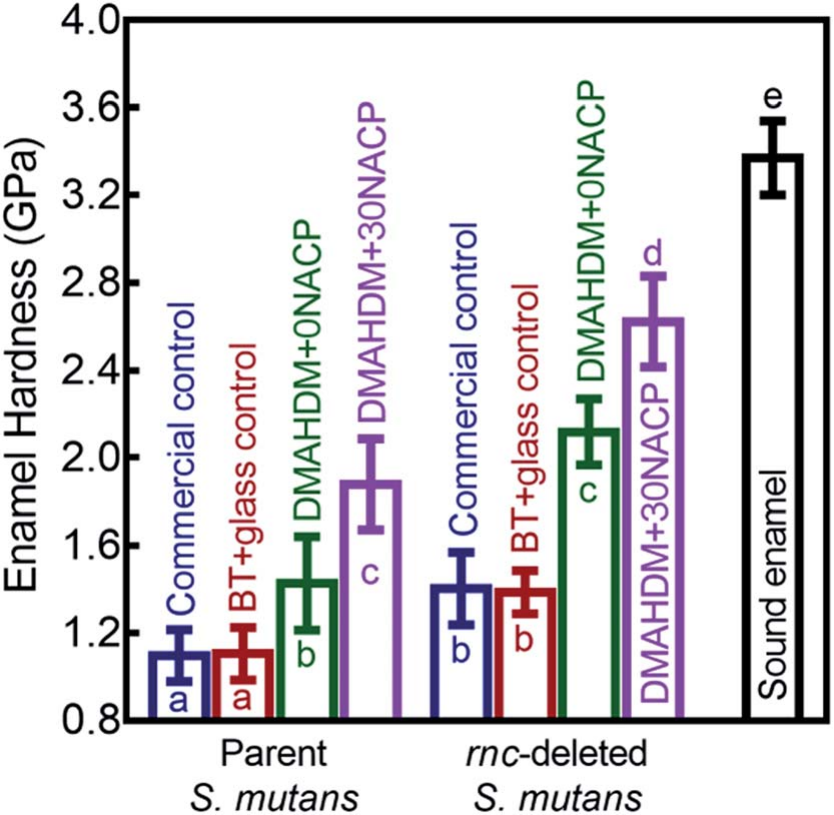
Comparisons of enamel hardness at the margins of composite restorations. Since the three distances from the margins had little effect on enamel hardness, for each group, the hardness values at the three distances were pooled together and averaged (mean ± sd; *n* = 108). Biofilm acid attacks for 30 days greatly reduced the enamel hardness for control groups. The combination of *rnc*-deletion with DMAHDM–NACP composite protected enamel under biofilms and yielded the greatest enamel hardness among all the groups. Values with dissimilar letters are significantly different from each other (*p* < 0.05).

Fig. 8 shows that the three distances from the margins had little effect on enamel hardness. This demonstrates that for the control, the biofilm acids demineralized the enamel all the way from 50 μm to 250 μm, and that the DMAHDM+30NACP protected the enamel all the way from 50 μm to 250 μm. Therefore, the enamel hardness values at the three distances were pooled together and averaged in Fig. 8 to have a more direct and simpler comparison between the groups (mean ± sd; *n* = 108). After 30 days of parent *S. mutans* biofilm acids, enamel hardness was reduced from 3.37 GPa to 1.10 GPa in control groups. The *rnc* deletion increased the enamel hardness by 27%, to 1.40 GPa in control groups. Remarkably, the combination of *rnc*-deletion and DMAHDM+30NACP composite yielded the greatest enamel hardness (*p* < 0.05), which was 2.4 times that of control. These results showed that: (1) the *rnc*-deletion in *S. mutans* substantially increased the enamel hardness, even with no DMAHDM or NACP; (2) the combination of *rnc*-deletion plus 3% DMAHDM (without NACP) increased the enamel hardness by 92%, compared to parent *S. mutans* with control groups; (3) the combination of *rnc*-deletion and DMAHDM+30NACP composite yielded the greatest enamel hardness, which was 140% higher than those of control groups.

## 4. Discussion

The present study used an *in vitro* secondary caries biofilm model and demonstrated that combining *rnc* gene-deletion in *S. mutans* with DMAHDM–NACP nanocomposite suppressed caries at the margins and protected enamel hardness for the first time. The hypotheses were proven that the *rnc* gene deletion in *S. mutans* greatly reduced the biofilm formation and polysaccharide synthesis. In addition, the novel DMAHDM–NACP composite substantially reduced the biofilm acid production. Most importantly, the dual strategy of *rnc* gene-deletion plus DMAHDM–NACP nanocomposite achieved the greatest reduction in biofilm growth and acid production, decreasing the biofilm CFU by 6 logs. Moreover, the combination of *rnc* gene-deletion with DMAHDM–NACP nanocomposite had the best protection for the restoration margins. This resulted in the greatest enamel hardness at the margins under biofilm acid attacks for 30 days among all the tested groups, yielding an enamel hardness that was 2.4-fold of commercial fluoride-releasing control composite. Therefore, the dual strategy of *rnc*-deletion + DMAHDM–NACP composite appeared to be highly promising for controlling recurrent caries at the margins, protecting the tooth structures and prolonging the restoration lifetime.

In the present study, the F ion release from Heliomolar showed no significant antibacterial activity. As reported previously, the bacterial growth and metabolism were affected by F ions; however, the F ion concentrations would need to exceed 526.3 μmol L^−1^ (or 10 ppm).^70^ The F ion release in the culture medium of Heliomolar was much less than 10 ppm.^71^ Therefore, the F ion release from Heliomolar showed no significant antibacterial activity, which was similar to the BT+glass control group in the present study. Polymerizable QAMs are promising for dental applications, including incorporations into composites, cements, sealants, primers and adhesives.^12,14,23^ DMAHDM can chemically bond in the polymer network, thus displaying long-term effects.^23,56^ In the present study, 3% mass fraction of DMAHDM in the composite did not negatively affect the flexural strength and the elastic modulus. The DMAHDM composite substantially reduced the biofilm metabolic activity and lactic acid and polysaccharide productions, and reduced the biofilm CFU by 4 orders of magnitude.

DMAHDM and NACP were combined into the composite. The Ca and P ion releases from the composite were greater at pH 4 than those at pH 7. This pH-triggered “smart” feature could greatly increase the ion release at a cariogenic pH condition when such ions would be the most needed to inhibit tooth demineralization and promote remineralization.^27^ In addition, when the cariogenic bacteria produced acids to demineralize the tooth, the NACP composite could neutralize the acid to increase the pH back to a safe pH region, to avoid demineralization of the enamel and tilt the balance toward remineralization.^29,30,72^ Therefore, the NACP–DMAHDM composite, with triple benefits of antibacterial, acid-neutralization, and remineralization capabilities, could contribute to inhibiting secondary caries around the restoration margins.

For effective antibacterial treatments, targeting the EPS matrix is recognized as an effective strategy to remove biofilms.^41^ Several other organisms (*e.g.*, non-*mutans streptococci*, Actinomyces and Lactobacillus species) can produce soluble glucans and/or fructans in the EPS.^73,74^ Polysaccharides produced by *S. mutans* are the main constituents in the matrix of cariogenic plaque biofilms recognized as an essential virulence factor.^73–75^ To this end, the regulating genes of EPS matrix formation are optimal targets for the development of anti-caries compounds.^40,41,73^ The *rnc* gene participates in the formation and virulence expression of *S. mutans* biofilm.^39,40^ Indeed, the present study demonstrated that the *rnc*-deleted *S. mutans* had significantly less cariogenicity with less biofilm polysaccharide and lactic acid productions, compared to the parent *S. mutans*. Furthermore, the *rnc* gene-deletion substantially increased the biofilm's penetrability by antibacterial agents and the drug-susceptibility of the biofilm, thus reducing the biofilm CFU by 6 orders of magnitude, compared to the control. Due to the meritorious results of the present study, we plan in a further study to investigate the gene modifications of other key cariogenic bacterial species, such as those in the oral candidiasis group, to determine their effects on dental caries prevention.

In the oral environment, dental plaque contains bacteria that ferment carbohydrates to various organic acids, which dissolve the dental hard tissues.^11^ In alternating time periods, this loss of tissues may be remineralizated by a deposit of minerals from saliva.^64,65^ These two processes cause the occurrence of the destruction (caries) or repair.^64,65^ When the balance is tilted toward demineralization, secondary caries eventually occurs in enamel along the tooth-restoration margins.^76^ In the demineralization process, *S. mutans* acts as the principle cariogenic bacteria; hence, several previous studies used *in vitro S. mutans* biofilm models to investigate the efficacy of various anticaries agents.^63–65^ In the present study, we grew *S. mutans* biofilms on tooth specimen surfaces for 30 days by using a bacterial demineralization model, including all steps of the caries process: bacterial acid formation, demineralization, and remineralization. According to previous studies, the 4 hours of duration in the BHIS led to results that were similar to the *in vivo* oral conditions with accumulated food cycling daily.^63,77,78^ To mimic the saliva composition, additional Ca and P ions were supplied in this model.^64,65^ In addition, the buffering effect of the PIPES in the growth medium was similar to that of the buffer in the whole saliva *in vivo*.^64,65^ Therefore, the biofilm model of the present study resembled to the oral environment regarding the demineralization and remineralization cycles per day, Ca and P ions, and buffering effects. The enamel hardness for the best group is still significantly lower than the hardness of sound enamel; this is likely due to the following reasons. First, the present study used a mono-species model of *S. mutans*. This is a cariogenic and accelerated model, producing a stronger demineralization effect than what happens clinically in the oral environment where there is a mixture of benign and cariogenic bacterial species.^79^ Second, BHI was included in the growth medium in this model. The biofilm could produce acid at all the times as an accelerated model. Third, no tooth brushing was performed in this model, while clinically tooth brushing is recommended once or twice daily using tooth pastes containing F ions. Further study is needed to investigate whether an experimental model more closely mimicking the clinical situation would result in an enamel hardness similar to that of sound enamel.

Indeed, this biofilm model effectively caused the enamel hardness at the margins to decrease to 1 GPa for the control group restored with a commercial fluoride-releasing control composite. This indicates that secondary caries would occur at the margins under such biofilm attacks. In sharp contrast, the novel dual strategy of *rnc*-deletion plus DMAHDM+30NACP composite greatly reduced the enamel demineralization at the margins under biofilms. The enamel hardness from the dual strategy was about 2.6 GPa, more than 2-fold of that in the commercial fluoride-releasing control group. This was likely due to the following factors. (1) The antibacterial effects from DMAHDM reduced the acid production from biofilms.^12,14,23,26^ (2) The NACP composite released Ca and P ions, especially at cariogenic pH, thereby promoting remineralization.^29,30,72^ Therefore, the novel DMAHDM–NACP nanocomposite displayed both remineralization and antibacterial capabilities, thus inhibiting secondary caries.^13,31^ (3) The deletion of *rnc* gene in *S. mutans* disrupted the biofilm growth and EPS formation.^39,40^ The less biofilm and EPS mass reduced the protection for bacteria, increased the killing efficacy of the DMAHDM, and enhanced the penetrability of agents, compared to parent *S. mutans* biofilm.^41,74^ Collectively, *rnc*-deletion plus DMAHDM–NACP composite were demonstrated in the present study to be highly beneficial for the prevention of secondary caries at the margins.

Regarding potential clinical applications, it would be highly advantageous to use targeted EPS treatment in dentistry to improve the antibacterial and remineralization efficacy of DMAHDM–NACP composites. Previous studies demonstrated the safety and efficacy of the *rnc*-deleted *S. mutans* in the oral cavity in an animal model for 50 days, and the inhibitory effects on dental caries occurrence was indeed achieved *in vivo*.^39,40^ Here the *rnc* gene-deletion can significantly reduce the biofilm matrix formation, and it in turn increases the killing potency of the DMAHDM. In addition, since the *rnc* gene-deletion can reduce the biofilm acid production, it enhances the remineralization efficacy of NACP, because NACP promote remineralization more strongly at a higher pH than at a lower pH. Therefore, the combination of DMAHDM–NACP composite with *rnc* deletion has exciting potential for clinical applications to inhibit secondary caries and protect tooth structures. Furthermore, several of the *rnc* gene-deletion or the RNase III post-transcriptional regulatory pathway inhibitors in *S. mutans* are worthy candidates for further investigation, with potential for development of drugs for alternative and complementary caries treatments. A compound delivering the inhibitors could potentially be applied after tooth cavity preparation, or could be coated onto enamel to reduce secondary caries and protect the tooth structures. Further studies are needed to use a multi-species biofilm model and a human saliva-based dental plaque model, and to investigate the novel combination of *rnc*-deletion plus DMAHDM–NACP nanocomposite in experiments that simulate the oral environment.

## 5. Conclusion

This study investigated the novel approach of combining *rnc* gene-deletion in *S. mutans* with DMAHDM–NACP nanocomposite to inhibit secondary caries at the restoration margins for the first time. The *rnc* gene-deletion in *S. mutans* greatly reduced the polysaccharide synthesis. The multifunctional DMAHDM+30NACP nanocomposite had remineralization and antibacterial capabilities, reducing the biofilm CFU by 4 logs. Remarkably, combining the *rnc* deletion in *S. mutans* with DMAHDM+30NACP composite achieved the most potent antibiofilm effect, reducing biofilm CFU by about 6 logs, compared to parent *S. mutans* on a commercial fluoride-releasing control composite. Under 30 days of biofilm acid attack, the dual strategy of *rnc*-deletion plus DMAHDM+30NACP composite reduced enamel demineralization, yielding 2.4 times greater enamel hardness than control near the margins. Therefore, the novel dual strategy of bacterial gene-modification and bioactive DMAHDM–NACP nanocomposite is promising to inhibit recurrent caries, protect tooth structures, and increase the longevity of dental restorations.

## Conflicts of interest

There are no conflicts to declare.

## Supplementary Material
